# Supplementary material for: Self-generated surface magnetic fields inhibit laser-driven sheath acceleration of high-energy protons
Source: Nat Commun. 2018 Jan 18;9:280. doi: 10.1038/s41467-017-02436-w (PMC5773560; doi:10.1038/s41467-017-02436-w)
Supplement: Supplementary file 1 — Supplementary Information [file 41467_2017_2436_MOESM1_ESM.pdf]

Supplementary Note 1: Correlation between regions of strong magnetic fields, electron depletion and proton deflection

Supplementary Figure 1 shows various field and particle distributions from a 2D particle-in-cell (PIC) simulation performed with the PICLS code for a laser intensity $I_{\mathrm{L}} = 6.6 \times 10^{20}\ \mathrm{W\,cm^{-2}}$, a pulse duration of 400 fs, and a wavelength $\lambda_{\mathrm{L}} = 0.5\ \mu\mathrm{m}$ (corresponding to a dimensionless field strength $a_0 = 11$). From the electron energy density (Supplementary Figure 1c), we observe that, in the population of electrons that are detached from the target (and which were accelerated early on in the laser pulse, before the B-field would reach its maximum strength), there is a strong depletion in the strong B-field regions [Supplementary Figure 1b]. We also observe that those electrons crossing the target rear in the late part of the laser pulse are trapped by the magnetic field along the target surface (see the detailed electron trajectories in Fig. 1 of the main text) and are prevented to move forward in the sheath. This disrupts the continuous supply of hot electrons which is crucial for TNSA acceleration to be efficient; by limiting the number of hot electrons within the expanding sheath, the magnetization quenches the ion accelerating electrostatic field earlier than would have taken place otherwise. Note that this is true for such relatively long laser pulses – Supplementary Notes 7 and 8 discuss the benefit brought in this respect by using shorter laser pulses.

The proton energy density displayed in Supplementary Figure 1d further reveals that the protons at the acceleration front are transversely (and hence angularly as well, see Supplementary Notes 2 and 3 for more details) modulated due to the magnetic field; being deflected outwards, they also move away from the high-sheath-field region, which reduces further the acceleration efficiency. This impresses on them a ring-like pattern that is well consistent with the experimental observations made at high intensity (see Fig. 4 of the main text).

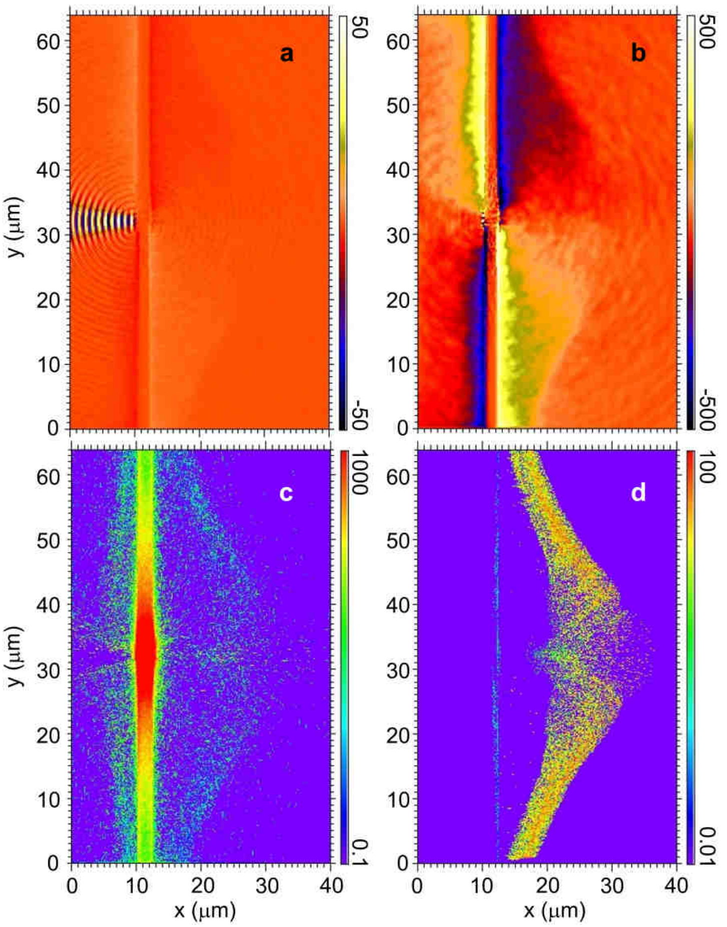

Supplementary Figure 1: Interaction of a tightly focused intense laser with a thin Al foil. 2D PICLS simulation of a laser pulse of 6.6×10^{20} W cm^{-2} intensity, 400 fs FWHM duration, 0.5 µm wavelength and 1.6 µm FWHM spot size interacting with a 2 µm thick Al target. **a** Laser electric field (in units of 6.4×10^{12} V m^{-1}). **b** Quasistatic magnetic field (in units of 10^{6} G). **c** Electron energy density (in units of $100n_{\mathrm{c}}$ keV, where $n_{\mathrm{c}} = 1.1 \times 10^{21}$ cm^{-3} is the critical density at 1 µm wavelength. **d** Proton energy density (in units of $100n_{\mathrm{c}}$ keV). All snapshots are taken 100 fs after the peak of the laser pulse.

Supplementary Note 2: Modeling particle trajectories inside expanding plasma fields

In complement to Fig. 1 of the main text, it is of interest to analyze the trajectories of electrons and protons inside the strong DC fields generated at the target surfaces. For this purpose, we consider a 2D xy domain, where the x-axis is taken to be parallel to the target rear normal (consistently with the simulations presented in the main text). The electromagnetic field is considered steady, with $\mathbf{E} = (E_x, 0, 0)$ and $\mathbf{B} = (0, 0, B_z)$. A relativistic treatment is considered for the particles, which do not interact between themselves.

The trajectory of a particle of charge q, mass m, position $\mathbf{x}$, velocity $\mathbf{v}$, momentum $\mathbf{p}$ and Lorentz factor γ is defined by the following equations of motion,

$$\frac{dp_x}{dt} = q\left(E_x + v_y B_z\right), \tag{1}$$

$$\frac{dp_y}{dt} = -q v_x B_z, \tag{2}$$

$$v_x = \dot{x} = \frac{p_x}{m\gamma}, \tag{3}$$

$$v_y = \dot{y} = \frac{p_y}{m\gamma}. \tag{4}$$

A particle trajectory starting at $t = t_0$ has as input parameters:

$$\mathbf{p}(t_0) = m_e c\sqrt{\gamma^2(t_0) - 1},$$
$$p_x(t_0) = p(t_0)\cos\theta(t_0),$$
$$p_y(t_0) = p(t_0)\sin\theta(t_0),$$

where we have introduced the initial Lorentz factors, $\gamma_e(t_0) = 1 + \epsilon_{\mathrm{e}}(t_0)[\mathrm{MeV}]/0.511$ for the electrons, and $\gamma_p = 1 + \epsilon_{\mathrm{p}}[\mathrm{GeV}]/0.94$ for the protons, where $\epsilon_{\mathrm{e,p}}$ denotes the kinetic energies. The initial angle with respect to the x axis is taken to be $\theta(t_0) = 0$. The initial velocities, $v_x(t_0)$ and $v_y(t_0)$, are used in Supplementary Eqs. (1) and (2) to yield $\boldsymbol{p}(t_1)$, $\theta(t_1)$ and $\gamma(t_1)$. Then, the particle position is advanced through $\boldsymbol{p}(t_1 - \Delta t)\Delta t/m\gamma(t_1)$. The time step Δt is chosen to be 0.02 fs for electrons and 0.1 fs for protons.

The B-field map shown in Fig. 1a,e of the main text is approximated in the form

$$B_z = -B_0(x)\sin\left(\frac{\pi}{2}\frac{y-y_0}{\Delta y_\mathrm{B}}\right)$$

for $|y-y_0| \le \Delta y_\mathrm{B}$, and in the form

$$B_z = -\mathrm{sgn}(y-y_0)B_0(x)\exp\left(-\frac{|y-y_0|-\Delta y_\mathrm{B}}{L_{y\mathrm{B}}}\right),$$

for $|y-y_0| > \Delta y_\mathrm{B}$. The longitudinal profile is assumed to be

$$B_0(x) = B_\mathrm{max}\exp[-(x-x_0)/L_{x\mathrm{B}}],$$

for $x \ge x_0$ and $B_0(x) = 0$ otherwise. To reproduce the PIC-simulated *B*-field distributions shown in Fig. 1a,e we set $x_0 = 10$ µm and $y_0 = 30$ µm, and use the best-fitting values detailed in Supplementary Table 1.

Supplementary Table 1: Best-fitting parameters for the PIC-simulated *B*-field distributions

	Fig. 1a<br>$I_\mathrm{L}\lambda_\mathrm{L}^2 = 6.5\times10^{19}$ W µm^{2}cm^{-2}<br>$\phi_\mathrm{L} = 1$ µm	Fig. 1e<br>$I_\mathrm{L}\lambda_\mathrm{L}^2 = 2\times10^{21}$ W µm^{2}cm^{-2}<br>$\phi_\mathrm{L} = 1.6$ µm
B_max	100 MG	500 MG
Δy_B	5 µm	6 µm
$L_{x\mathrm{B}}$	4 µm	5 µm
$L_{y\mathrm{B}}$	20 µm	20 µm

Supplementary Table 2: Best-fitting parameters for the PIC-simulated E-field distributions

	Fig. 1.a1<br>$I_\mathrm{L}\lambda_\mathrm{L}^2 = 6.5\times10^{19}$ W µm^{2}cm^{-2}<br>$\phi_\mathrm{L} = 1$ µm	Fig. 1.b1<br>$I_\mathrm{L}\lambda_\mathrm{L}^2 = 2\times10^{21}$ W µm^{2}cm^{-2}<br>$\phi_\mathrm{L} = 1.6$ µm
E_max	1 TV m^{-1}	3 TV m^{-1}
Δx_E	0.5 µm	0.5 µm
Δy_E	2 µm	2 µm
L_{xE}	4 µm	5 µm
L_{yE}	20 µm	20 µm

As for the E-field, it is taken in the form

$$E_x(x,y) = E_0(x)$$

for $|y-y_0| \le \Delta y_\mathrm{E}$, and in the form

$$E_x(x,y) = E_0(x)\exp\left(-\frac{|y-y_0|-\Delta y_{\mathrm{E}}}{L_{y\mathrm{E}}}\right)$$

for $|y-y_0| > \Delta y_{\mathrm{E}}$. The longitudinal profile is assumed to be

$$E_0(x) = E_{\max}$$

for $x \le x_0 + \Delta x_{\mathrm{E}}$, and

$$E_0(x) = E_{\max}\exp[-(x - x_0 - \Delta x_{\mathrm{E}})/L_{x\mathrm{E}}]$$

otherwise. Moreover, $E_0(x) = 0$ is assumed inside the target ($x < x_0$). The parameters best fitting the simulated E-field results are given in Supplementary Table 2.

Typical particle trajectories are shown in Supplementary Figure 2 ($I_{\mathrm{L}}\lambda_{\mathrm{L}}^2 = 6.5 \times 10^{19}$ W μm^{2}cm^{-2}, $\phi_{\mathrm{L}} = 1$ μm, $B_{\max} = 100$ MG) and Supplementary Figure 3 ($I_{\mathrm{L}}\lambda_{\mathrm{L}}^2 = 2 \times 10^{21}$ W μm^{2}cm^{-2}, $\phi_{\mathrm{L}} = 1.6$ μm, $B_{\max} = 500$ MG), which correspond to Fig. 1a and Fig. 1e of the main text, respectively. For $B_{\max} = 100$ MG (Supplementary Figure 2), electrons of energies in the 10 MeV range suffer strong magnetic deflections, yet still move in the forward direction ($x > 0$). By contrast, protons of a few MeV energies are weakly influenced by the magnetic field. For $B_{\max} = 500$ MG (Supplementary Figure 3), the proton trajectories are strongly deflected so that a hollow pattern forms in their spatial distribution. As a result, one of the outstanding features of TNSA protons, namely their high laminarity, is ruined. As for the electrons, their forward motion is severely hampered, most of them being trapped at the target rear surface (consistently with the PIC simulations shown in Supplementary Figure 1).

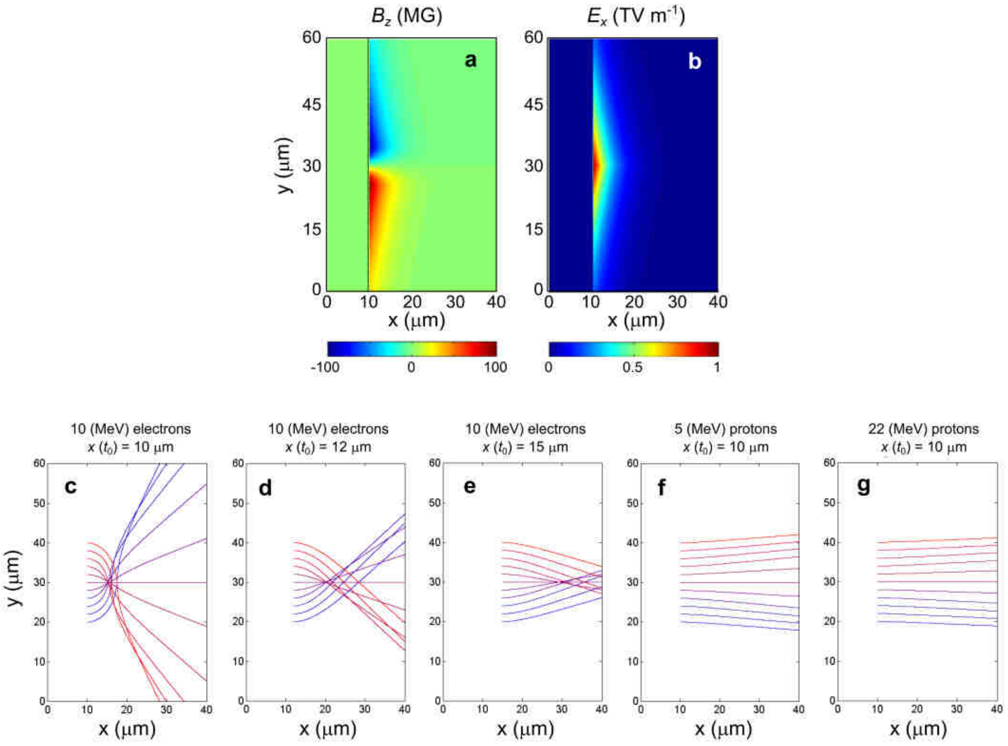

Supplementary Figure 2: Model-predicted fields and particle trajectories at a 6.5×10^{19} W μm^{2}cm^{-2} intensity. **a,b** B_z (in MG) and E_x (in TV m^{-1}) field maps reproducing the PIC simulation results at an intensity of 6.5×10^{19} W μm^{2}cm^{-2} (see Fig. 1a of the main text). The parameters are $B_{\max} = 100$ MG and $E_{\max} = 1$ TV m^{-1}. **c-e** Trajectories of 10 electrons starting from different initial transverse positions $[\,y(t_0) - y_0 = -10, -8, -6, \ldots + 10$ μm$\,]$ around the symmetry axis $y = y_0$. The initial kinetic energy of the electrons is 10 MeV and they are injected along $x > 0$ at various locations: (**c**) $x(t_0) = x_0$, (**d**) $x(t_0) = x_0 + 2$ μm and (**e**) $x(t_0) = x_0 + 5$ μm. **f-g** Trajectories of 10 protons starting from $x = x_0$ and different initial y positions ($y(t_0) - y_0 = -10, -8, -6, \ldots + 10$ μm). The initial kinetic energies of protons are (**f**) 5 MeV and (**g**) 22 MeV, and they are injected along $x > 0$. The symmetry axis is positioned at $y_0 = 30$ μm and $x_0 = 10$ μm is the location of the target rear surface.

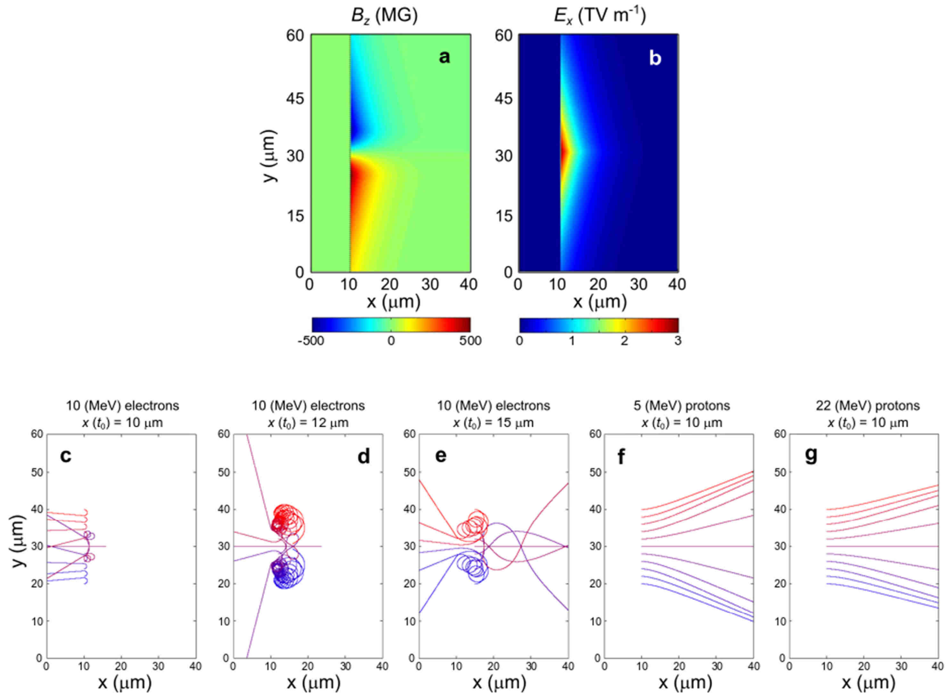

Supplementary Figure 3: Model-predicted fields and particle trajectories at a 2×10^{21} W $\mu m^2 cm^{-2}$ intensity. a, b B_z (in MG units) and E_x (in TV m^{-1} units) maps reproducing the PIC simulation results at an intensity of 2×10^{21} W $\mu m^2 cm^{-2}$ (see Fig. 1e of the main text). The parameters are and $B_{max} = 500$ MG and $E_{max} = 3$ TV m^{-1}. **c-e** Trajectories of 10 electrons starting from different initial transverse positions $[y(t_0) - y_0 = -10, -8, -6, \ldots + 10\ \mu m]$ around the symmetry axis $y = y_0$. The initial kinetic energy of the electrons is 10 MeV and they are injected along $x > 0$ at various locations: (**c**) $x(t_0) = x_0$, (**d**) $x(t_0) = x_0 + 2\ \mu m$ and (**e**) $x(t_0) = x_0 + 5\ \mu m$. **f-g** Trajectories of 10 protons starting from $x = x_0$ and different initial y positions $(y(t_0) - y_0 = -10, -8, -6, \ldots + 10\ \mu m)$. The initial kinetic energies of protons are (**f**) 5 MeV and (**g**) 22 MeV, and they are injected along $x > 0$. The symmetry axis is positioned at $y_0 = 30\ \mu m$ and $x_0 = 10\ \mu m$ is the location of the target rear surface.

Supplementary Note 3: Angle-energy proton distributions

The peaks of the angular distributions measured by the RCF at a laser intensity of $I_{\mathrm{L}}\lambda_{\mathrm{L}}^2 = 1.3 \times 10^{21}$ W μm^2 cm^{-2} and a spot size of $\phi_{\mathrm{L}} = 1.6$ μm (Figs. 4f-h of the main text) exhibit strong dependency on the proton energy. This is summarized in Supplementary Figure 4a, on which is overlaid the angle-energy distribution extracted from the 2D PICLS simulation (red dots). Both experimental and simulated distributions show two distinct proton groups, indicated by lines 1 and lines 2. Line 1 (resp. 2) corresponds to a population of protons with deflection angle increasing (resp. decreasing) with energy. Moreover, the average magnetic field, $\langle B_z \rangle$, seen by protons of a given energy ϵ_{p} as inferred from their measured deflection angle via the relation $\theta = e\langle B_z \rangle l/m_{\mathrm{p}}v_{\parallel}$, is plotted in Supplementary Figure 4a with open blue squares. We have defined $v_{\parallel} = \sqrt{2\epsilon_{\mathrm{p}}/m_{\mathrm{p}}}$ as the longitudinal proton velocity, and l is the longitudinal extent of the magnetized region. We take $l \sim 5$ μm as suggested by the simulated *B*-field map displayed in Fig. 1e in the main text.

The PIC-simulated angular distributions of the protons are further detailed in Supplementary Figure 4b,c for low- and medium-energy protons. These angular distributions are observed to be quite consistent with the experimental data shown respectively in Fig. 4.g,h,j,k of the main text

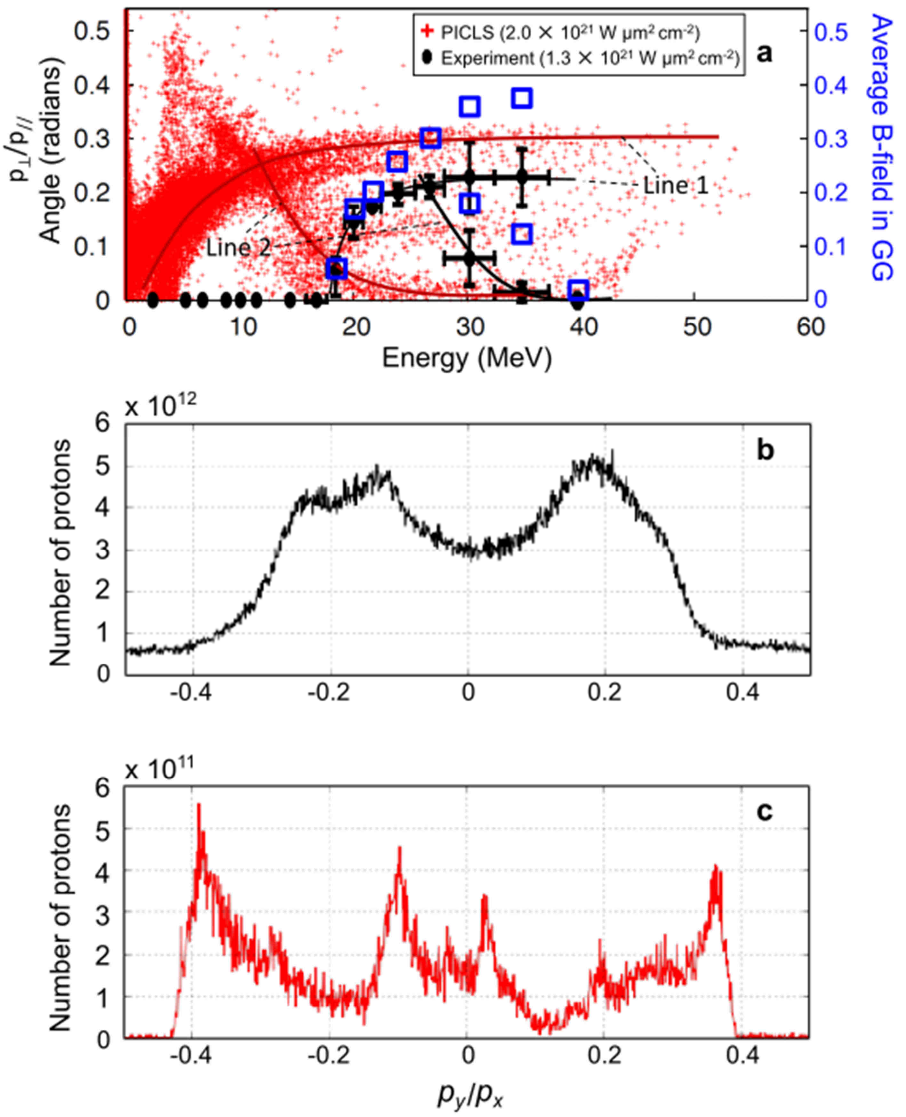

Supplementary Figure 4: Angle-energy proton distributions. a Red small dots are from a 2D PICLS simulation performed at a 2×10^{21} W μm^2 cm^{-2} intensity (see Fig. 4.b of the main text), measured 300 fs after the laser peak. Large black dots are experimental results obtained at similar intensity (see Fig. 4f-h of the main text). Blue open squares are the average magnetic field (in GG) extracted from the experimentally observed angular deflection (see text in detail). **b** Angular distribution of low-energy protons ($<$ 10 MeV), obtained from PICADOR simulations performed with the same parameters as in (**a**). **c** Same as (**b**) for mid-energy protons ($10 - 20$ MeV).

95

Supplementary Note 4: Experimental scalings of the maximum proton energy

The plot shown in Supplementary Figure 5is an update of Fig. 4 of Ref. [1]. The plot compiles data from the following references: (i) [2,3,4,5,6,7,8,9,10,11,12,13] for laser pulse durations between 30 and 100 fs; (ii) [14,15,16,17,18,19,20,21,22,23] for pulse durations between 100 fs and 1 ps; (iii) [24,25] for pulse durations above 1 ps.

The dispersion in the data for a given laser intensity can be attributed to different target and laser parameters (notably, the prepulse level). However, this compilation displays the same trend as in Ref. [2] between ultra-short laser pulses (< 100 fs) and longer pulses: below 10^{19} W μm^2 cm^{-2}, the proton energies obtained with ultra-short pulses (< 100 fs) are clearly below those achieved with longer pulses, while they progressively become of the same order, and possibly higher, above 10^{20} W μm^2 cm^{-2}.

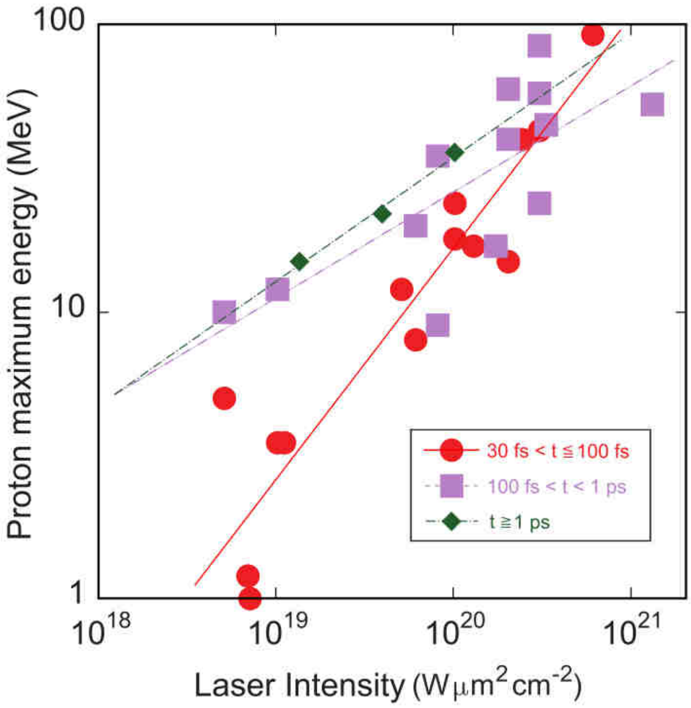

Supplementary Figure 5: Maximum proton energy vs. on-target laser intensity. Compilation of experimental data obtained using various laser facilities and grouped according to the laser pulse duration (denoted “t” in the legend).

Supplementary Note 5: Proton acceleration in the ultra-high-intensity ultra-short-pulse interaction regime

In Supplementary Figure 6are shown additional PIC simulations, the aim of which is to show that, for few-cycle laser pulses interacting with nanometer-size foils, no significant B-field develops on the target surfaces and that the radiation-pressure mechanism is able to accelerate ions (here H^+ and C^{6+}) to very high energies, in contrast to the simulations presented in the rest of the paper, which employed longer laser pulses and thicker targets.

These 2D simulations were performed using the CALDER code [26]. The simulation domain has dimensions $30000\Delta x \times 8000\Delta y$ with mesh sizes $\Delta x = 1.3$ nm and $\Delta y = 6.4$ nm. The laser pulse, of 0.8 μm wavelength, is Gaussian in space and time, with 1.9 μm spot size and 15 fs duration (FWHM). The peak intensity is either $I_\mathrm{L} = 5 \times 10^{21}\ \mathrm{W\,cm^{-2}}$ or $I_\mathrm{L} = 1.8 \times 10^{22}\ \mathrm{W\,cm^{-2}}$. The target is a 100 nm-thick, fully ionized carbon foil at solid density, coated on both front and rear sides with a Δx-thick H^+ layer. The initial electron and ion temperatures are $T_\mathrm{e} = T_\mathrm{i} = 10$ eV. Collisions and radiation losses are not described. 500 particles are used per cell and species.

The above interaction conditions correspond to the relativistic-transparency regime which is known to optimize ion acceleration [27], and which translates into both significant laser absorption ($\sim 20 - 25\%$) and reflection ($\sim 30 - 60\%$) rates. In this regime, ion acceleration results from the combined action of radiation pressure and rear-side sheath field. Maximum proton energies of 120 MeV and 250 MeV are reached for $I_\mathrm{L} = 5 \times 10^{21}\ \mathrm{W\,cm^{-2}}$ and $1.8 \times 10^{22}\ \mathrm{W\,cm^{-2}}$, respectively. Because the electrons keep on being accelerated (reaching mean energies of $\sim 5 - 10$ MeV for the two intensities considered) as part of the laser pulse shines through the foil, they are less affected by the magnetostatic field, whose peak strength is measured to be in the $0.5 - 1$ GG range when proton acceleration starts saturating. Consequently, the hot-electron density does not exhibit any depletion off axis, in contrast to what is observed in Supplementary Figure 1. Also, the B-field peaks quite far away transversely from the central region where the ion acceleration takes place, causing the ion density patterns shown in Supplementary Figure 6c,d,g,h (evolving into mushroom-shaped profiles at later times).

140

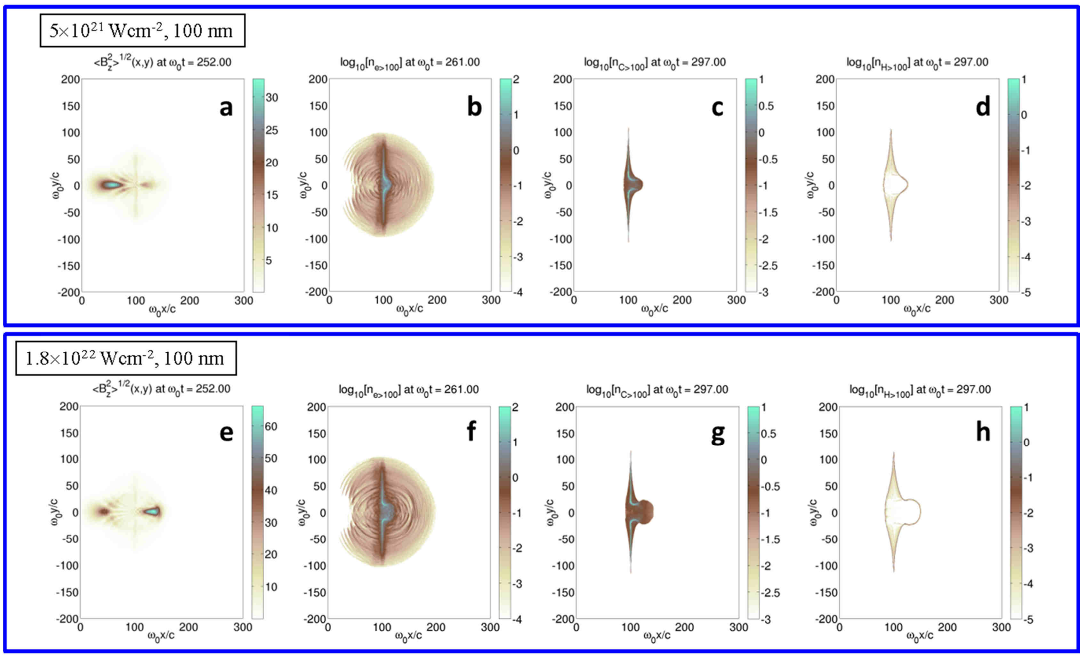

Supplementary Figure 6: Proton acceleration from sub-micron targets driven by ultra-high-intensity ultra-short lasers. 2D CALDER PIC simulation of the interaction of 15 fs laser pulses of intensity (**a-d**) $I_{\mathrm{L}} = 5 \times 10^{21}\ \mathrm{W\,cm^{-2}}$ and (**e-g**) $I_{\mathrm{L}} = 1.8 \times 10^{22}\ \mathrm{W\,cm^{-2}}$ with a $100\ \mathrm{nm}$-thick carbon foil. **a**,**e** Quadratic average of the B-field, $\langle B_z^2 \rangle^{1/2}$ (in units of $100\ \mathrm{MG} = 10^4\ \mathrm{T}$, note that the strong peak corresponds to the laser field). **b**,**f** Density (in units of $10^{21}\ \mathrm{cm^{-3}}$) of electrons of kinetic energies $> 100\ \mathrm{keV}$. **c**,**g** Density of carbon ions of kinetic energies $> 100\ \mathrm{keV}$. **d**,**h** Density of protons of kinetic energies $> 100\ \mathrm{keV}$. Space and time coordinates are in units of $c/\omega_0 = 0.16\ \mu\mathrm{m}$ and $\omega_0^{-1} = 0.53\ \mathrm{fs}$, respectively ($\omega_0$ is the angular laser frequency). The on-target peak intensity is reached at $t = 205\omega_{\mathrm{L}}^{-1}$. The title of each figure indicates the time at which it is recorded. The ion density maps are shown at the saturation time for ion acceleration.

141

Supplementary Note 6: Model-predicted laser dependence of the *B*-field strength

Supplementary Figure 7 displays the variation of the *B*-field with the laser spot size, as predicted by our 1-D model. To calculate it, we first introduce the characteristic time, t_{char}, when the expanding protons have moved a distance equal to the local Debye length. From the model, this characteristic time appears to be about twice the inverse plasma ion frequency, ω_{pi}^{-1}. Supplementary Figure 7b shows that t_{char} depends on the laser intensity, but also the laser spot size. This is due to the fact that, for a given hot electron divergence (a $45°$ half angle is used here) and target thickness ($2\ \mu\text{m}$), hot electron dilution occurs faster at smaller spot sizes. Supplementary Figure 7a explains how the lateral spread of the hot electrons due to recirculation is estimated at $t = t_{\text{char}}$, yielding the sheath diameter, $2r$. Then, the magnetic field strength, $B_z(t)$, follows from time integrating the sheath electric field $E_x(t)$ divided by the sheath radius r. Supplementary Figure 7d plots the *B*-field strength at the laser intensity peak, $B_z(t_{\text{max}})$. Note that its maximum value is limited to $B_{\text{max}} \equiv \left(2\mu_0 n_{\text{h,rear}} k_{\text{B}} T_0\right)^{1/2}$. It appears that moderate spot sizes ($\phi_{\text{L}} = 4 - 10\ \mu\text{m}$) give slightly stronger *B*-fields than at tight focusing ($\phi_{\text{L}} = 0.9\ \mu\text{m}$). Only when the spot size becomes comparable with the sheath size, does the *B*-field start to decrease.

Overall, this implies that the magnetic inhibition highlighted in our present study, using tight focusing in order to access high laser intensities, will likely be even stronger under more standard focusing conditions (e.g. $\phi_{\text{L}} = 4 - 10\ \mu\text{m}$).

Supplementary Figure 8 illustrates the model-predicted dependency of the *B*-field against the laser pulse duration. Although shorter pulse durations lead to smaller magnetic fields (due to the finite growth rate of the *B*-field, see also further discussion in Supplementary Note 7), strong magnetic fields of few 100 MG are predicted for laser intensities $> 10^{21}\ \text{W cm}^{-2}$, even for 50 fs duration pulses. However, Supplementary Figure 8b shows that the electron magnetization (quantified by the ratio of the electron Larmor radius and the acceleration front), and therefore the magnetic inhibition of proton acceleration, is reduced when shortening the pulse duration down to $\tau_L = 50$ fs. In short, this clearly shows the benefit of using laser shorter pule durations to lessen the magnetic inhibition highlighted in our study.

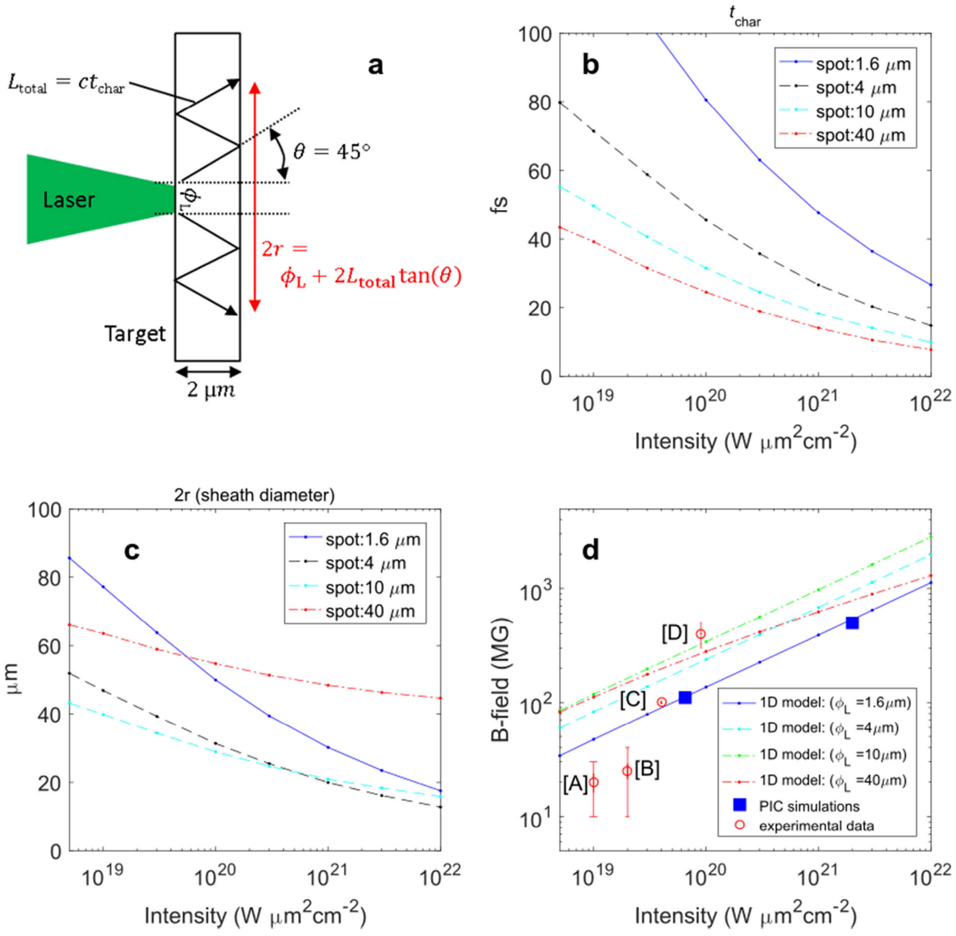

Supplementary Figure 7: Model-predicted variation of the *B*-field strength with the laser spot size. a Schematic drawing to show the lateral spread of the hot electrons due to recirculation is estimated at $t = t_{\text{char}}$, yielding the sheath diameter, $2r$. **b** Characteristic time t_{char} when the plasma expansion starts, defined as the time when the front protons have moved a distance equal to the local Debye length. **c** Sheath diameter at $t = t_{\text{char}}$ due to fast electron recirculation and transverse spread. **d** Magnetic field strength calculated for various laser spot sizes and laser peak intensities, taken at the laser intensity peak. For all curves, the laser wavelength is $\lambda_L = 1$ μm. The red circles correspond to experimental observations ([A] G. Sarri et al., Phys. Rev. Lett. **109**, 205002 (2012), [B] B. Albertazzi et al., Phys. Plasmas **22**, 123108 (2015), [C] W. Schumaker et al., Phys. Rev. Lett. **110**, 015003 (2013), [D] M. Tatarakis et al. Nature **415**, 280 (2002)), while the blue boxes come from the PIC simulations of the paper (see Fig.1 of the main text).

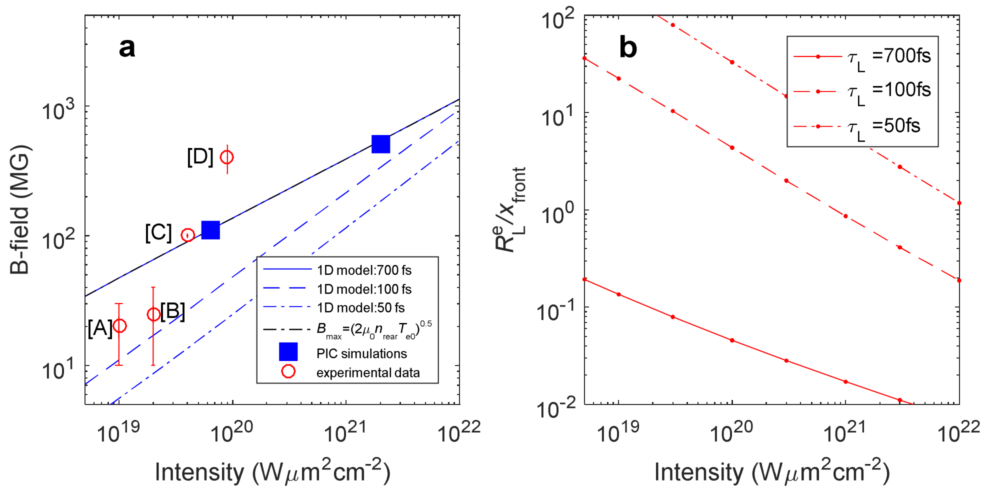

Supplementary Figure 8: Model-predicted variations of the *B*-field strength and electron magnetization level with the laser pulse duration. a *B*-field (in MG) at the laser intensity peak. **b** Normalized electron Larmor radius $R_{\mathrm{L}}^{\mathrm{e}}/x_{\mathrm{front}}$ at the laser intensity peak. The laser spot size and wavelength are $\phi_{\mathrm{L}} = 1.6$ µm and $\lambda_{\mathrm{L}} = 1$ µm, respectively.

173

Supplementary Note 7: Proton acceleration and electron magnetization with a 50 fs laser pulse

Supplementary Figure 9 displays the results of a PIC simulation run with a laser pulse of duration (50 fs) much shorter than that used in the highest-intensity case discussed in the main text (800 fs, at SNL). The other laser parameters were kept equal to those used in Fig. 1e of the main text (i.e., $I_{\mathrm{L}} = 2 \times 10^{21}\ \mathrm{W\,\mu m^2 cm^{-2}}$, $\phi_{\mathrm{L}} = 1.6\ \mu\mathrm{m}$ and $\lambda_{\mathrm{L}} = 1\ \mu\mathrm{m}$). This simulation, performed with the PICADOR code (see Methods), aims to show that the reduction in the acceleration timescale that results from a shorter laser pulse weakens the magnetization effects on proton acceleration. When comparing the simulation results with 50 fs and 700 fs pulse durations, several points can be noticed: (i) In the 50 fs case, the generated magnetic field at the peak of the laser pulse is the same order. (ii) Similar electron energies are reached (which was rather expected since the laser intensities are the same). (iii) Similar maximum proton energies are attained, although a slightly higher value is found for a 50 fs pulse, consistent with previous researches (Supplementary Figure 5).

Since in the 50 fs case, the laser intensity reaches its maximum much quicker, the protons reach their saturation energy over a much shorter distance, and hence the sheath longitudinal length is shorter than for a 700 fs pulse. It is in fact even shorter than the electron Larmor radius over the timescale of proton acceleration, so that the electrons are weakly magnetized in the 50 fs case, even though the magnetic field strength are similar for both pulse durations (compare Supplementary Figure 9c and Fig. 1h in the main text).

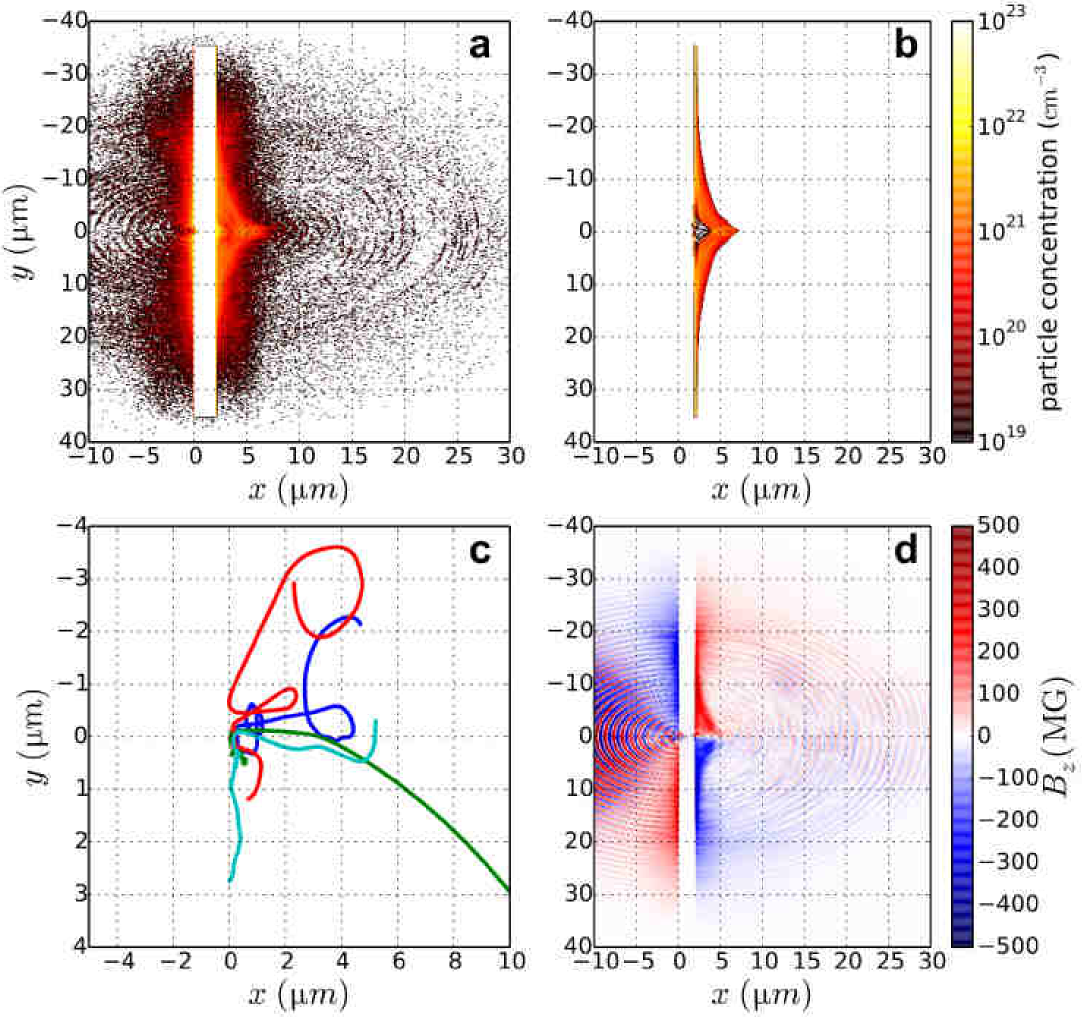

Supplementary Figure 9: Proton acceleration at high intensity by a 50 fs laser pulse. 2D PICADOR PIC simulation of a 50 fs laser pulse of intensity $I_\mathrm{L} = 2 \times 10^{21}$ W μm^{2}cm^{-2}, spot size $\phi_\mathrm{L} = 1.6$ μm and wavelength $\lambda_\mathrm{L} = 1$ μm interacting with a 2 μm thick Al foil coated with a 20 nm thick proton layer on its rear side. Shown are the (**a**) electron and (**b**) ion densities (in cm^{-3}), (**c**) trajectories of sampled energetic electrons, and (**d**) the transverse magnetic field, B_z (in MG). All snapshots are taken at the laser intensity peak.

Supplementary Note 8: Proton acceleration and electron magnetization in a reduced mass target

In Supplementary Figure 10 are shown the results of an additional PICADOR PIC simulation performed with a small-width target (i.e., a so-called reduced mass target, RMT). The laser parameters are those used in Fig. 1e-h in the main text, *i.e.*, $\tau_L = 700$ fs, $\phi_L = 1.6$ μm, $I_L = 2 \times 10^{21}$ W μm^{2}cm^{-2}. The target is similar to that considered in Fig. 1 except that its transverse width is reduced to 20 μm. The purpose of this simulation is to demonstrate that proton acceleration in RMTs is still affected by magnetization effects.

In this case, it is seen that the transverse gradients of the magnetic field on the target rear side are smaller than when using a large target (as in Fig. 1 of the main text). However, the field itself is comparable in strength and topology with that in a large target. This may contradict the intuitive view that reducing the target transverse size makes the electron sheath flatter, thus reducing the inductive *B*-field generated by the transverse gradient of the sheath electric field via $\partial B_z/\partial t \propto \partial E_x/\partial y$. However, the magnetic field is also produced by $\partial E_y/\partial x$ gradients, which can be greater in RMTs. Indeed, whilst the longitudinal scales are then close to those found in large targets, the transverse electric field is somewhat greater due to denser hot electrons.

Generally speaking, the *B*-field generation at the target rear side follows from the hot electrons expelled by the laser pulse. They form an electric current on axis, which is actually the original source of the magnetostatic field. The electric fields involved in the time-dependent Faraday law can be seen as an intermediate step between the current and the magnetic field, which determine the steady growth of the magnetic field up to the stage when the magnetic pressure becomes comparable with the plasma pressure. From simulations, we estimated the current intensity and the magnitude of the magnetic field and found that, close to the target rear surface, they are consistent with the quasistatic Ampere's law $\nabla \times \mathbf{B} = \mu_0 \mathbf{J}$. The current intensity, in turn, depends only on the number and velocities of the laser-driven hot electrons, both depending on the laser intensity and target density, rather than on the target geometry. This is the reason why the *B*-field strength is weakly sensitive to the transverse target width. This magnetic field is strong enough to magnetize electrons as can be seen from the electron

trajectories shown in Supplementary Figure 10c. As a consequence, it acts detrimentally on proton acceleration (through the same mechanisms as described in the manuscript). This explains why RMTs yield only a modest increase in proton energies compared to large targets and why, even at very high intensities ($> 10^{21}$ W μm^{2}cm^{-2}), they do not give rise to the proton acceleration level predicted by the simple 1D, unmagnetized model.

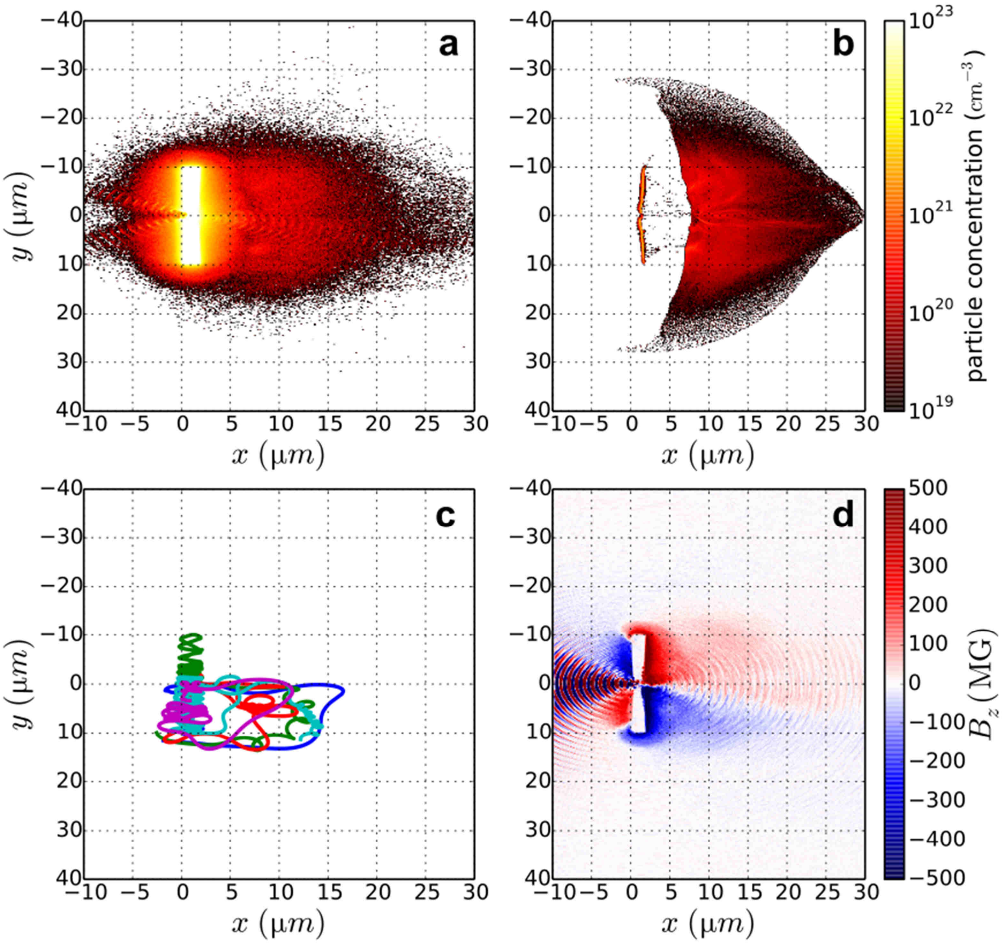

Supplementary Figure 10: Proton acceleration at high laser intensity in a reduced mass target. 2D PICADOR PIC simulation of a 700 fs laser pulse of intensity $I_{\mathrm{L}} = 2 \times 10^{21}$ W μm^{2}cm^{-2}, spot size $\phi_{\mathrm{L}} = 1.6$ μm and wavelength $\lambda_{\mathrm{L}} = 1$ μm interacting with an Al foil of 2 μm thickness and 20 μm transverse size, coated with a 20 nm thick proton layer on its rear side. Shown are the (**a**) electron and (**b**) ion densities (in cm^{-3}), (c) trajectories of sampled energetic electrons, and (**d**) the transverse magnetic field, B_z (in MG). All snapshots are taken at the laser intensity peak.

Supplementary Note 9: Sensitivity of ellipsoid plasma mirror focusing to plasma expansion

The peak laser intensity on the ellipsoid plasma mirror (EPM) surface was about $3 \times 10^{14} - 5 \times 10^{14}$ W cm^{-2} for both the LULI and SNL experiments. This yielded a cumulated fluence on the plasma mirror surface of $F \simeq 130 - 180$ J cm^{-2} around the pulse maximum, and of $F \simeq 280 - 370$ J cm^{-2} at the end of the pulse (*i.e.*, at $t = \tau_L$ after the laser intensity peak). The time-dependent plasma temperature T_e at the critical-density surface of the $\mathrm{SiO_2}$ material composing the EPM approximately varies as $T_\mathrm{e}(\mathrm{eV}) \simeq F(\mathrm{J\ cm^{-2}})$ [28]. This simple scaling allows one to infer the expansion of the critical-density surface, as shown in Supplementary Figure 11. The expansion length is predicted to be of 0.07 μm around the laser peak, and of 0.19 μm at the end of the pulse.

Ray-trace simulations were performed to assess the effect of this surface expansion on the focusing quality of the EPM (Supplementary Figure 12). Within the $f/4$ laser cone incoming into the EPM, we selected 71 rays with a 0.2° angular step. The plasma was assumed to expand normally to the EPM surface. The ray-trace calculations reveal that the plasma expansion is too weak to degrade significantly the final spot size. Even for a much larger ($> 10\,\times$) expansion length (2 μm), the spot size at the second focus of the EPM is significantly smaller than the diffraction limited size. Hence, for our laser parameters, the surface expansion of the EPM is not expected to affect our results.

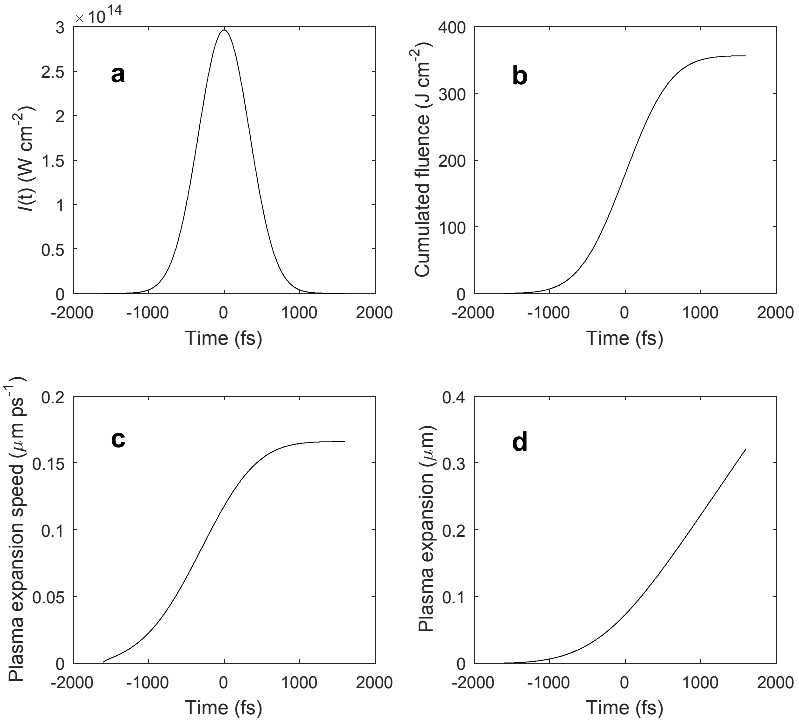

Supplementary Figure 11: Predicted expansion of the critical-density surface of the EPM. The laser parameters are those of the highest-fluence SNL experiment: $90\,\mathrm{J}$ on-target laser energy, $1\,\mu\mathrm{m}$ wavelength, $800\,\mathrm{fs}$ duration and the beam diameter on the plasma mirror surface is $5/\cos\theta$ mm, with $\theta = 39°$. The expansion length is predicted to be of $\sim 0.07\,\mu\mathrm{m}$ around the laser peak and $\sim 0.19\,\mu\mathrm{m}$ at the end of the pulse, i.e., when the laser intensity reaches $I(t = 800\,\mathrm{fs}) \sim 0.06 I_\mathrm{L}$. Shown are the temporal evolutions of (**a**) the laser intensity on the EPM surface, **(b)** cumulated fluence (in $\mathrm{J\,cm^{-2}}$), **(c)** expansion velocity of the critical-density surface (in $\mu\mathrm{m\,ps^{-1}}$) and (**d**) plasma expansion length (in µm). The expansion velocity is estimated to be $c_\mathrm{s} = \sqrt{Z_\mathrm{eff} T_\mathrm{e}/A m_\mathrm{p}}$ with A the atomic mass, m_p the proton mass and an ionization state $Z_\mathrm{eff}\,/\,Z = 0.7$, assuming all the electrons except the 1s and 2s of Si and O are ionized. The electron temperature at the expanding surface is supposed to vary as T_e (eV) $\approx F(\mathrm{J\,cm^{-2}})$ [28].

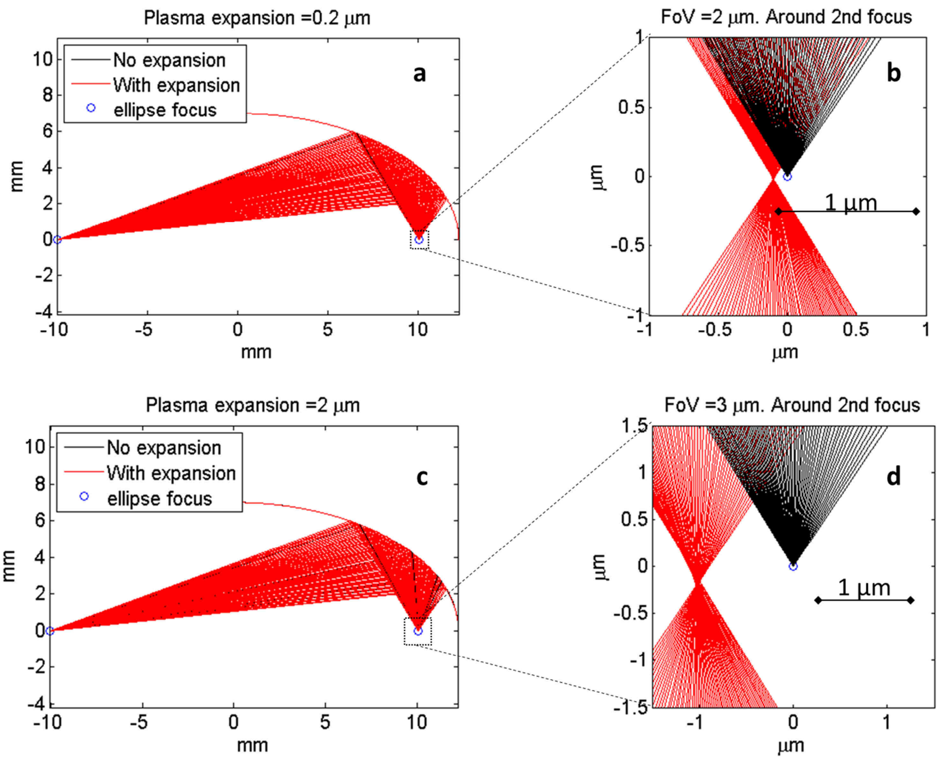

Supplementary Figure 12: Ray-tracing simulation of the EPM focusing in the geometrical optics approximation. In (**a**, **b**), the reflection surface is shifted by 0.2 μm along the local normal of the ellipsoid to take into account the surface expansion. **b** is a zoom of (**a)** around the second focus of the ellipsoid. The resulting focal spot is, however, significantly smaller than the diffraction-limited spot size (~ 1 μm). Black rays are reference rays in the absence of plasma expansion, thus they focus to an infinitively small spot. In (**c**, **d**) an exaggerated 2 μm expansion length is considered. The resulting spot size is still smaller than 1 μm, which demonstrates the robustness of the EPM against plasma expansion during the pulse. In all cases, the incident rays are fixed.

257

SUPPLEMENTARY REFERENCES

1 Macchi, A., Borghesi, M., and Passoni, M. Ion acceleration by superintense laser-plasma interaction. *Reviews of Modern Physics* **85**, 751-793 (2013).

2 Spencer, I. , Ledingham, K. W. D., McKenna, P., McCanny, T., Singhal, R. P., Foster, P. S., Neely, D., Langley, A. J., Divall, E. J., Hooker, C. J., Clarke, R. J., Norreys, P. A., Clark, E. L., Krushelnick. K., and Davies, J. R., Experimental study of proton emission from 60-fs, 200-mJ high-repetition-rate tabletop-laser pulses interacting with solid targets, *Phys. Rev. E* **67**, 046402 (2003).

3 Fritzler, S. *et al.*, Proton beams generated with high-intensity lasers: Applications to medical isotope production, *App. Phys. Lett.* **83**, 3039-3041 (2003).

4 Fuji, T. *et al.*, MeV-order proton and carbon ion acceleration by irradiation of 60 fs TW laser pulses on thin copper tape, *App. Phys. Lett.* **83**, 1524-1526 (2003).

5 Mackinnon, A. J. *et al.*, Enhancement of Proton Acceleration by Hot-Electron Recirculation in Thin Foils Irradiated by Ultraintense Laser Pulses, *Phys. Rev. Lett.* **88**, 215006 (2002).

6 Oishi, Y. *et al.*, Dependence on laser intensity and pulse duration in proton acceleration by irradiation of ultrashort laser pulses on a Cu foil target, *Phys. Plasmas* **12**, 073102 (2005).

7 Zeil, K., Kraft, S. D., Bock, S., Bussmann, M., Cowan, T. E., Kluge, T., Metzkes, J., Richter, T., Sauerbrey, R., and Schramm, U., The scaling of proton energies in ultrashort pulse laser plasma acceleration, *New J. Phys.* **12**, 045015 (2010).

8 Ogura, K. *et al.*, Proton acceleration to 40 MeV using a high intensity, high contrast optical parametric chirped-pulse amplification/Ti:sapphire hybrid laser system, *Optics Letters* **37**, 2868-2870 (2012).

9 Burdonov, K. F., Eremeev, A. A., Ignatova, N. I., Osmanov, R. R., Sladkov, A. D., Soloviev, A. A., Starodubtsev, M. V., Ginzburg, V. N, Kuz'min, A. A., Maslennikova, A. V., Revet, G., Sergeev, A. M., Fuchs, J., Khazanov, E. A., Chen, S., Shaykin, A. A., Shaikin, I. A., and Yakovlev, I. V., Experimental stand for studying the impact of laser-accelerated protons on biological objects, *Quantum Electronics* **46**, 283-287 (2016).

10 Ceccotti, T., Lévy, A., Popescu, H., Réau, F., D’Oliveira, P., Monot, P., Geindre, J. P., Lefebvre, E., and Martin, Ph. Proton Acceleration with High-Intensity Ultrahigh-Contrast Laser Pulses, *Phys. Rev. Lett*. **99**, 185002 (2007).

11 Kim, I. J., Pae, K. H., Choi, I. W., Lee, C. -L., Kim, H. T., Singhal, H., Sung, J. H., Lee, S. K., Lee, H. W., Nickles, P. V., Jeong, T. M., Kim, C. M., and Nam, C. H. Radiation pressure acceleration of protons to 93 MeV with circularly polarized petawatt laser pulses, *Phys. Plasmas* **23**, 070701 (2016).

12 Dollar, F., Zulick, C., Thomas, A. G. R., Chvykov, V., Davis, J., Kalinchenko, G., Matsuoka, T., McGuffey, C., Petrov, G. M., Willingale, L., Yanovsky, V., Maksimchuk, A., and Krushelnick, K. Finite Spot Effects on Radiation Pressure Acceleration from Intense High-Contrast Laser Interactions with Thin Targets, *Phys. Rev. Lett.* **108**, 175005 (2012).

13 Henig, A., Steinke, S., Schnürer, M., Sokollik, T., Hörlein, R., Kiefer, D., Jung, D., Schreiber, J., Hegelich, B. M., Yan. X. Q., Meyer-ter-Vehn, J., Tajima, T., Nickles, P. V., Sandner W., and Habs, D. Radiation-Pressure Acceleration of Ion Beams Driven by Circularly Polarized Laser Pulses, *Phys. Rev. Lett.* **103**, 245003 (2009).

14 Murakami, Y. *et al.*, Observation of proton rear emission and possible gigagauss scale magnetic fields from ultra-intense laser illuminated plastic target, *Phys. Plasmas* **8**, 4138-4143 (2001).

15 McKenna, P., Ledingham, K. W. D., Yang, J. M., Robson, L., McCanny, T., Shimizu, S., Clarke, R. J., Neely, D., Spohr, K., Chapman, R., Singhal, R. P., Krushelnick, K., Wei, M. S., and Norreys, P. A. Characterization of proton and heavier ion acceleration in ultrahigh-intensity laser interactions with heated target foils, *Phys. Rev. E* **70**, 036405 (2004).

16 Maksimchuk, A. *et al.*, High-energy ion interactions by short laser pulses, *Plasma Phys. Reports* **60**, 473 (2004).

17 Snavely, R. A. *et al.*, Intense High-Energy Proton Beams from Petawatt-Laser Irradiation of Solids, *Phys. Rev. Lett.* **85**, 2945-2948 (2000).

18 Fuchs, J., Antici, P., D'Humieres, E., Lefebvre, E., Borghesi, M., Brambrink, E., Cecchetti. C. A., Kaluza, M., Malka, V., Manclossi, M., Meyroneinc, S., Mora, P., Schreiber, J., Toncian, T., Pepin, H., and Audebert, P. Laser-driven proton scaling laws and new paths towards energy increase, *Nat. Phys.* **2**, 48-54 (2006).

19 Bartal, T., Foord, M. E., Bellei, C., Key, M. H., Flippo, K. A., Gaillard, S. A., Offermann, D. T., Patel P. K., Jarrott, L. C., Higginson, D. P., Roth, M., Otten, A., Kraus, D., Stephens, R. B., McLean, H. S., Giraldez, E. M., Wei, M. S., Gautier D. C., and Beg, F. N. Focusing of short-pulse high-intensity laser-accelerated proton beams, *Nat. Phys*. **8**, 139–142 (2012).

20 Wagner, F., Deppert, O., Brabetz, C., Fiala, P., Kleinschmidt, A., Poth, P., Schanz, V. A., Tebartz, A., Zielbauer, B., Roth, M., Stöhlker, T., and Bagnoud, V. Maximum Proton Energy above 85 MeV from the Relativistic Interaction of Laser Pulses with Micrometer Thick CH2 Targets, *Phys. Rev. Lett.* **116**, 205002 (2016).

21 Henig, A., Kiefer, D., Markey, K., Gautier, D. C., Flippo, K. A., Letzring, S., Johnson, R. P., Shimada, T., Yin, L., Albright, B. J., Bowers, K. J., Fernandez, J. C., Rykovanov, S. G., Wu, H. -C., Zepf, M., Jung, D., Liechtenstein, V. Kh., Schreiber, J., Habs, D., and Hegelich, B. M. Enhanced

Laser-Driven Ion Acceleration in the Relativistic Transparency Regime, *Phys. Rev. Lett.* **103**, 045002 (2009).

22 Kar, S., Kakolee, K. F., Qiao, B., Macchi, A., Cerchez, M., Doria, D., Geissler, M, McKenna, P., Neely, D., Osterholz, J., Prasad, R., Quinn, K., Ramakrishna, B., Sarri, G., Willi, O., Yuan, X. Y., Zepf, M., and Borghesi, M. Ion Acceleration in Multispecies Targets Driven by Intense Laser Radiation Pressure, *Phys. Rev. Lett.* **109**, 185006 (2012).

23 Hornung, M., Keppler, S., Liebetrau, H., Kessler, A., Seidel, A., Hellwing, M., Schorcht, F., Körner, J., Sävert, A., Polz, J., Becker, G., Arunachalam, A. K., Klöpfel, D., Hein, J., and Kaluza, M. C. Status of the POLARIS laser system, 8th workshop on High-Energy-Class Diode-Pumped Solid-State Lasers, Oxford, 26th March 2014, http://www.clf.stfc.ac.uk/clf/resources/pdf/talk_1.pdf.

24 Spencer, I., Ledingham, K. W. D., Singhal, R. P., McCanny, T., McKenna, P., Clark, E. L., Krushelnick, K., Zepf, M., Beg, F. N., Tatarakis, M., Dangor, A. E., Norreys, P. A., Clarke, R. J., Allott, R. J., and Ross, I. N. Laser generation of proton beams for the production of short-lived positron emitting radioisotopes, *Nucl. Instrum. Meth. B*, **183**, 449-458 (2001).

25 Zepf, M., Clark, E. L., Krushelnick, K., Beg, F. N., Escoda, C., Dangor, A. E., Santala, M. I. K., Tatarakis, M., Watts I. F., Norreys, P. A., Clarke, R. J, Davies, J. R., Sinclair M. A., Edwards, R. D., Goldsack, T. J., Spencer, I., and Ledingham K. W. D. Fast particle generation and energy transport in laser-solid interactions, *Phys. Plasmas* **8**, 2323-2330 (2001).

26 Lefebvre, E. *et al.* Electron and photon production from relativistic laser–plasma interactions. *Nucl. Fusion* **43**, 629-633 (2003).

27 Esirkepov, T., Yamagiwa, M., and Tajima, T. Laser Ion-Acceleration Scaling Laws Seen in Multiparametric Particle-in-Cell Simulations. *Phys. Rev. Lett.* **96,** 105001 (2006).

28 Doumy, G. *et al*. Complete characterization of a plasma mirror for the production of high-contrast ultraintense laser pulses. *Phys. Rev. E* **69**, 026402 (2004).
